# Epidermal nevus: a rare presentation of congenital epidermal hamartoma in a young female

**DOI:** 10.11604/pamj.2025.51.104.47800

**Published:** 2025-08-25

**Authors:** Anshika Kishor Singh, Gaurav Rajendra Sawarkar

**Affiliations:** 1 Department of Rachana Sharir, Mahatma Gandhi Ayurved College Hospital and Research Centre, Datta Meghe Institute of Higher Education and Research, Wardha, India

**Keywords:** Nevus sebaceus, inflammatory linear verrucous epidermal nevus (ILVEN), acanthosis nigricans

## Images in medicine

A 22-year-old female visited the outpatient department at Mahatma Gandhi Ayurved College Hospital and Research Centre in Salod (H), Wardha, with a skin lesion on her right breast that had been present for the past two years. She reported no previous treatment, no family history of similar skin conditions, and only occasional itching without any noticeable changes in the size or appearance of the lesion. Clinical examination revealed a linear arrangement of darkened, raised papules that had merged into a rough, plaque-like formation, following the embryonic Blaschko´s lines. The lesion was non-tender, and there were no signs of inflammation or systemic involvement. Based on its presentation, possible conditions such as nevus sebaceus, inflammatory linear verrucous epidermal nevus (ILVEN), and acanthosis nigricans were considered. However, the absence of inflammation and the pattern of the lesion supported a diagnosis of epidermal nevus. Although a skin biopsy was not performed, the visual and clinical features were strongly indicative. Epidermal nevi are non-cancerous skin growths arising from localized overproliferation of epidermal cells, often due to genetic mosaic mutations occurring during embryonic development. These lesions typically appear early in life but may go unnoticed or develop slowly, as seen in this case. Histopathological findings in such conditions usually show thickened skin layers and surface projections without gland involvement. While benign, these nevi can occasionally be linked with broader syndromic conditions or show changes later in life. This case is important not only for its clinical presentation in a young adult but also for the insight it offers into a rare skin condition within the context of integrative and ayurvedic medical learning.

**Figure 1:**
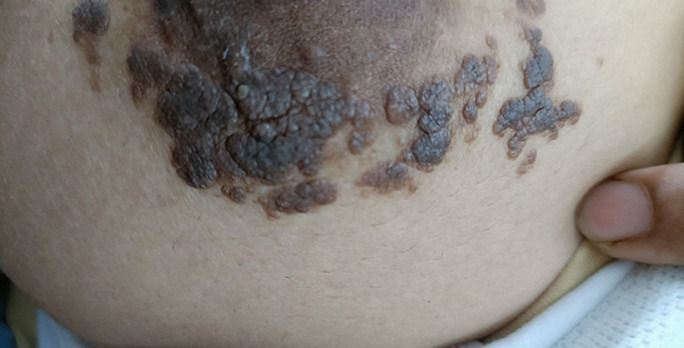
epidermal nevus presenting as a linear, hyperpigmented, verrucous plaque over the right breast

